# Self-Sensing Pneumatic Compressing Actuator

**DOI:** 10.3389/fnbot.2020.572856

**Published:** 2020-12-11

**Authors:** Nan Lin, Hui Zheng, Yuxuan Li, Ruolin Wang, Xiaoping Chen, Xinming Zhang

**Affiliations:** ^1^School of Data Science, University of Science and Technology of China, Hefei, China; ^2^School of Computer Science and Technology, University of Science and Technology of China, Hefei, China; ^3^School of Information Science and Technology, University of Science and Technology of China, Hefei, China

**Keywords:** soft robotics, design, safety, pneumatic actuator, self-sensing

## Abstract

Using soft pneumatic actuator is a feasible solution in the complex unstructured environment, owing to their inherent compliance, light weight, and safety. However, due to the limitations of soft actuators' materials and structures, they fall short of motion accuracy and load capacity, or need large-size, bulky compressors. Meanwhile, in order to gain better control, it is essential for them to sense the environments as well. This leads to high-price sensors or a complicated manufacture technique. Here, a self-sensing vacuum soft actuation structure is proposed, aiming at acquiring good balance among precision, output force, and actuation pressure. The actuator mainly comprises a flexible membrane and a compression spring. When actuated, the flexible membrane outside the actuator compresses the internal spring skeleton, realizing large contractile motion in axial direction. Its built-in force sensor can indirectly measure the absolute displacement of the actuator with certain accuracy (about 5% F.S.). Besides, it does not require high actuation pressure to generate enough output force. The actuator is quite easy to manufacture with low cost, and there are a variety of materials to choose from. We established quasi-static models for actuators built of two different kinds of membrane materials, and tested their accuracy and output force. In addition, to break through the limits of vacuum actuation, a method of positive-negative pressure combined actuation has been proposed, which lowers the requirements for air source equipments, increases actuation pressure, and reduces potential safety threats at the same time. This kind of soft actuators can also effectively resist and detect impacts. The design of a two-finger dexterous robot hand and robot joint based on this soft actuator illustrates its broad application prospects in the fields of mobile robots, wearable devices, and human–robot interaction.

## 1. Introduction

Soft actuators, relative to rigid mechanical structures, have been widely used in rescue, medical care, wearable devices (Ilievski et al., 2011; Kim et al., 2013; Cianchetti et al., 2014; Park et al., 2014; Rus and Tolley, 2015), etc, owing to their inherent compliance and safety. Traditional actuators like electric motors can reach high precision and speed, which makes it excel at repetitive tasks in industry. But they are often bulky and stiff, and a structured environment is needed for operation, otherwise they may do damage to the environment or break themselves. Soft actuators, by contrast, can perfectly adapt to the complex or dynamic situation, which reduces the threat to users.

Recently, research on soft actuator are promoted with the rapid development of flexible materials, structures, and sensors. According to the actuation method, there are mainly several kinds, that is, electromagnetic, thermal, chemical, fluid actuation, and hydraulic actuation.

Shape memory alloys (SMAs) (Jani et al., 2014), actuated by electric heating, have large contraction force output and the strain is also significant, yet their high non-linearity and hysteries are barriers to application. Similar to SMAs, shape memory polymers (SMPs) (Ahn et al., 2008; Hu et al., 2012) used more kinds of stimuli like chemical or light, while the response time is limited accordingly. Dielectric elastomer actuators (DEAs) (O'Halloran et al., 2008; Anderson et al., 2012), powered by high electric field, can meet the demands of high-frequency actuation. DEAs use electrostatic force to attract two different potential electrodes on either side of a compressible membrane, thus get large strain. Furthermore, Keplinger et al. proposed a novel hydraulical-electrostatic hybrid actuator, Peano-HASEL (Acome et al., 2018; Kellaris et al., 2018), based on Peano fluidic muscle (Sanan et al., 2014). It provided direct coupling of electrostatic and hydraulic forces for high-power and precise operation. The main remaining hurdle in using electrostatic actuation is the need of driving voltages up to the order of kilovolts, which is difficult to achieve and might be a potential safety issue, limits the usefulness of this technique.

Soft pneumatic actuators (SPAs) are the most popular actuators in soft robotics. SPAs make use of compressed air (or vacuum) as power source, so it will not cause any pollution to environment. Pneumatical driving offers other advantages such as lightweight, compliance, and inherent safety. The well-known McKibben artificial muscle, invented and developed in the 1950s (Gavrilović and Marić, 1969; Chou and Hannaford, 1996), is a landmark pneumatic actuator. The McKibben actuator comprises a rubber inner tube covered with a shell of braided, inextensible fibers. When the inner tube is inflated by positive pressure, the muscle swells radially and contracts axially to shorten its overall length. By using these flexible materials, the McKibben actuator considerably is more compliant and lightweight than common pneumatic cylinder. However, the contraction ratio is not very satisfactory (≤40%), and usually high pressure is required for operation. Several improvements have been preformed, such as choosing superior shell materials, structures, or implementing different membrane composition (Daerden and Lefeber, 2001; Villegas et al., 2012; Belding et al., 2018; Terryn et al., 2018), but high driving pressure is still essential. Contrary to McKibben muscle, fiber-reinforced actuators lengthen when pressurized (Galloway et al., 2013; Connolly et al., 2015, 2017). The actuators consist of a core bladder reinforced with inextensible fibers, which wrap around to limit the radial expansion. They are able to realize a wide range of motions (bending, twisting, and extension), and have larger strain (300% in Hawkes et al., 2016). However, the fabrication process for fiber-reinforced actuators is complicated.

Another famous SPA is pneumatic network (PneuNet) originally developed by Harvard University (Sun et al., 2013; Mosadegh et al., 2014). It is made almost entirely out of soft materials such as the silicone rubber, with a series of channels and chambers inside an elastomer. When pressurized, its channels are inflated and create assigned motion like bending or twisting. The PneuNets actuators are entirely soft, and can be inflated with low pressure, which guarantees the safety for human interaction and environmental adaptability. However, the inherent compliance also severely limits the stiffness and output forces, and makes it unstable.

In recent years, a novel design pattern combining origami with other actuation method has been attracting wide attention, and many outstanding achievements have emerged (Onal et al., 2013; Mu et al., 2015; Paez et al., 2016; Miyashita et al., 2017; Kim et al., 2018). Martinez et al. presented composite structures comprising elastomers and paper (or other flexible sheets) (Martinez et al., 2012). They fabricated the 3D paper structure using origami or laser cutting, and embedded it into silicone elastomers. Due to the self-folding character, this structure was able to reach high stretching ratio, and the sheet inside could reinforce the elastomeric matrix to withstand external disturbance. Yi et al. proposed a fiber-reinforced origamic robotic actuator (FORA) to improve the performance of McKibben-type artificial muscles by replacing the rubber inner tube with specially designed origamic chamber (Yi et al., 2018). Li et al. presented an architecture for fluidic artificial muscles, which could be programmed to produce complex multiaxial motion (Li et al., 2017). The fluid-driven origami-inspired artificial muscles (FOAMs) consisted of a compressible skeleton and a non-strechable membrane. When driven by negative pressure, the membrane deformed inwards to push the skeletal structure contract. This innovative structure made FOAMs extremely lightweight, low cost, and provided large contracting ratio.

In order for precise control, it is necessary to pair the actuator with sensors to create a feedback control system. But the deformable characteristic of soft robots prevents the use of many conventional sensors, and the accurate sensor models are often unavailable for calculation and analysis (Polygerinos et al., 2017). In soft robotics, alternative sensing methods with low-modulus sensors are preferred. Ionic liquid–based resistive sensors (Chossat et al., 2013; Yeo et al., 2016) are perceived by monitoring the resistance variation of the driven fluid. The elastomer layers of these sensors are often patterned with microfluidic channels, which are filled with liquid conductors (Majidi et al., 2011; Wong et al., 2012). liquid–based resistive sensors can be tuned by modulation of channel geometries, and are able to measure various types of strains (Vogt et al., 2013), thus have acquired wide attention in applications like soft robotic hand (Wall et al., 2017), wearable devices (Kramer et al., 2011b), and human fingers (Kramer et al., 2011a). But they suffer the large temperature drift due to the correlation between temperature and ion concentration. Besides, poor long-term stability and risk of leakage are also tricky. In other related works, conductive thermoplastic material is adopted to avoid these problems (Culha et al., 2014).

Capacitive sensors measure the capacitance variations caused by geometry changes when the elastic body is deformed. In these systems, dielectric layer is sandwiched between conductive soft plates, and conductive elements are employed to create conformable electrodes in the sensing system. Some electrostatic actuators like DEAs, EAPs, or Peano-HASEL naturally sense the deformation through capacitance monitoring (Jung et al., 2008; Kruusamäe et al., 2015; Acome et al., 2018) through their actuation mechanism. For other soft robots, nanowires (Lipomi et al., 2011), nanotubes (Amjadi et al., 2014), carbon black (Tsouti et al., 2016), and conductive fabrics (Atalay et al., 2017) are also used as dielectric layers. capacitive-based sensors offer some advantages over other systems, such as high linearity and fast response time, which are important parameters when the sensors are intended to be used in real-life scenarios (Lipomi et al., 2011; Hu et al., 2013). But they are sensitive to environmental contaminants, like proximity effect to conductive objects, and are mostly prone to cracking and delamination over extended usage of the sensor.

More recently, optical-based sensing has emerged as another soft sensor category where motion is detected through changes in the light that is emitted and received in a light guide (Zhao et al., 2016a; Harnett et al., 2017; Teeple et al., 2018). The probed optical signal properties can be intensity (Polygerinos et al., 2011), phase (Pang et al., 2007), frequency (Zook et al., 2000), or polarization (Saad et al., 1995). Optical-based sensors are insensitive to any environmental interference, thus already been used for tactile sensing in prosthetic fingers (Du et al., 2017), soft surgical manipulator (Sareh et al., 2014), and other clinical practices (Liu et al., 2011). Fiber optic intensity modulation is a common method that refers to a class of sensing techniques, and has been applied in a soft bending actuator (Zhao et al., 2016a) to detect motion and infer the actuator shape. Furthermore, the integration of stretchable optical waveguides makes the sensor seamlessly deform with the actuator (Zhao et al., 2016b), but these methods are limited by the assumption that the sensor curvature is uniform. Recently, other researchers have also explored optoelectronic shape detection with fiber Bragg gratings (FBGs), which reflect light with a peak wavelength that shifts in proportion to variations in strain and temperature (Hill and Meltz, 1997). Multiple FBGs can be fabricated on different longitudinal positions of one fiber to monitor the distributed strain and pressure (Zhuang et al., 2018). FBGs show great potential to develop completely soft strain sensors for soft continuum robots (Wang et al., 2016b), but expensive and complex fabrication process is the main barrier. Instead, TacTip sensors (Cramphorn et al., 2016; Wardcherrier et al., 2017, 2018) directly use embedded camera to monitor the deformation of a soft structure's skin, but rigid camera system is needed to integrate in soft robots.

Besides the aforementioned works, other soft sensors such as inductive (Rahimi et al., 2014; Felt et al., 2016), magnetic (Ozel et al., 2015; Wang et al., 2016a; Luoming et al., 2017), and piezoresistive sensors (Yamamoto et al., 2007; Shapiro et al., 2014) are also able to be put in practice. More details about soft sensing can be found in literatures (Wang et al., 2018). But until now, the production process of most soft sensors is intricate, especially hard to be integrated with the manufacture of actuators, or they may affect the movement of actuators.

In this paper, we present a novel linear actuator Self-sensing Pneumatic Compressing Artificial Muscle (SPCAM) based on the work of Li et al. (2017), but there are several key improvements. Driven by vacuum pressure, the actuator can realize the axial contraction motion, similar to muscle tissue. Besides, the actuator can be stretched passively, and the elastic structure ensures its safety and its impact resisting property. A simple but effective sensor, which has certain accuracy, is integrated into SPCAM without disturbing its motion, so SPCAM has the ability of self-sensing. The actuator is lightweight with large strain and output force. It is easy to manufacture and be put into mass production. Furthermore, a positive–negative pressure combined mechanism is proposed. That is, the SPCAM actuator can be embedded in other positive pressure actuation structures to lower the requirements of actuation pressure and enhance its accuracy, which makes it more practical in mobile devices.

The article is organized as follows: the design and actuating principles are presented in section 2. The membrane materials, SPCAM's static model among traction force, actuating pressure, and structure parameters, are analyzed in section 3. Its performance like accuracy and actuation force is tested in section 4. We combine the SPCAM with air cylinder and McKibben artificial muscle, which is presented in section 5. Finally, in sections 6 and 7, a two-finer dexterous gripper and a revolute joint for exoskeleton are designed to demonstrate SPCAM's great potential for applications; meanwhile, conclusions and future work are summarized.

## 2. Actuator Concept and Design

### 2.1. Schematics and Operation Principle of SPCAM

**Figure 1:**
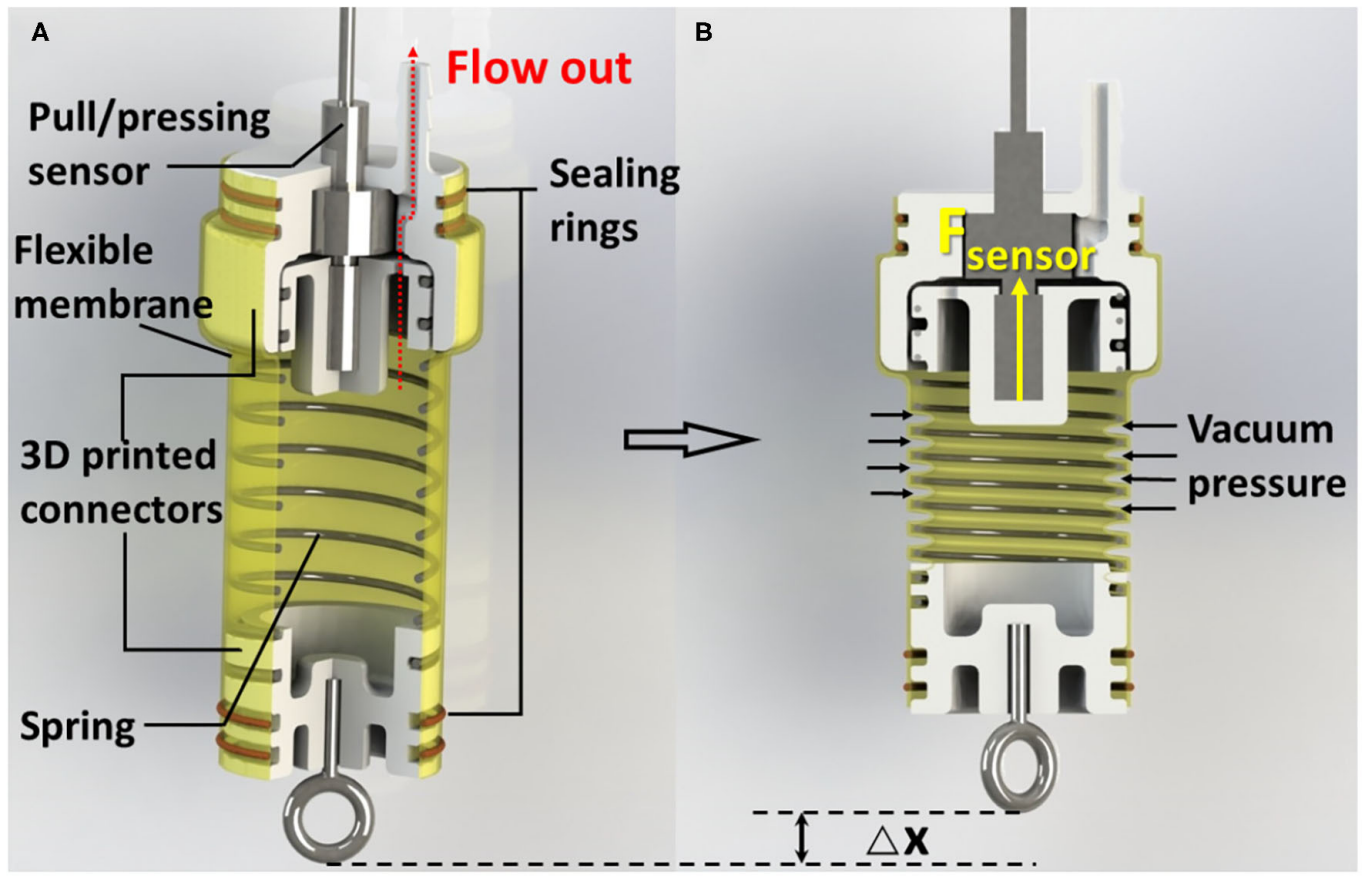
**(A)** Cutaway view of the Self-sensing Pneumatic Compressing Artificial Muscle (SPCAM). A typical SPCAM mainly consists of a compressing spring, 3D-printed parts, a flexible membrane, seals, and a pull/pressing sensor. **(B)** SPCAM under working state. The vacuum pressure pushes the membrane deform inward to make the actuator contract.

Figure 1 shows the schematic of SPCAM. The actuator consists of several major parts as follows: a spring skeleton, a flexible membrane outside, 3D-printed connectors, sealing rings, and a pull-pressure microsensor. The spring is sealed in the membrane, forming a compressible internal cavity. When the actuation pressure is zero, the self-locking performance is guaranteed by the elasticity of latex membrane and spring to some extent. Once we pump air out of the cavity, the pressure difference between internal and external part makes the membrane deform inward, similar to the work described in Li et al. (2017). The deformation of the membrane will exert pressure on the spring and makes it compress axially, generating a compressing force. The pulling/pressing sensor fixed on the end of the actuator is connected to the end of the spring, being able to sense the compressing force *F*_*sensor*_ (Figure 1B). Since both the spring and the pulling/pressing microsensor are inside of the cylindrical hyperelastic membrane, the outer pressure force and tension can be considered as external forces. According to Hooke's law, the compressing force scales linearly with respect to the displacement of the spring, so the displacement of the spring, that is, the displacement of the actuator, can be approximately calculated as the compressing force *F*_*sensor*_ divided by Hooke's coefficient.

### 2.2. Materials and Fabrication

The low cost of SPCAM is ensured by the standardized parts and simple assembly technology without any complicated procedure or advanced equipment (displayed in Supplementary Video). The core component is a flexible membrane and there are a variety of membrane materials to choose from. Here, we choose stretchable latex membranes and non-stretchable low-density polyethylene (LDPE) films for test. Other components, such as springs (304 stainless steel) and the pull-pressure microsensors (freud, DHMH-106, 1% F.S, 3 kg max.), are widely used in robots and automation equipment. There is an axial torque generated during contraction process, so we attach a microrotating ring to compensate the twist. The only non-standard part is 3D-printed connectors on the two ends, which can be replaced by injection molding parts if put into quantity production. The weight of the whole actuator is <40 g, 60% of which is occupied by the sensors, and it can be further reduced if the Micro Electromechanical System (MEMS) technology is adopted.

We have compared SPCAM with other four kinds of flexible actuators, taking their maximum strain and output force into consideration. Peano-HASEL (Kellaris et al., 2018) uses both electrostatic and hydraulic principles to linearly contract on application of voltage in a muscle-like fashion. PneuNet (Sun et al., 2013) is made with soft material and inner chambers. When pressurized, the inflated chambers will create assigned motion. FORA (Yi et al., 2018) is a fiber-reinforced origamic actuator, which improves the performance of McKibben-type artificial muscles. PPAM (Terryn et al., 2018) is another pneumatic artificial muscle whose membrane is constructed out of self-healing polymer.

Table 1 shows the comparison of five actuators. Here, the data of Peano-HASEL is acquired under the condition of 10 kV voltage. For other four actuators, the actuation pressure is limited with 40 kPa. The data show that SPCAM outperforms others in maximum output force and maximum strain. In the same time, our SPCAM design does not require customized parts and advanced fabrication techniques; while Peano-HASEL requires specialized high-voltage electrodes, others require complicated production procedure like pouring forming, which leads to higher cost.

**Table 1:** Comparison of five actuators.

**Actuator**	**SPCAM**	**Peano-HASEL**	**PneuNet**	**FORA**	**PPAM**
Maximum strain(%)	71	18	50	50	12
Maximum force(N)	40	10	2.2	50	18
Production complexity	Easy	Hard	Middle	Hard	Easy
Production cost	Low	Middle	Middle	High	Middle

## 3. Modeling and Analysis

### 3.1. Outer Membrane Material

We have chosen two different kinds of membrane materials to build the actuator. The first one is polyethylene membrane, as used in Li et al. (2017). It is a kind of non-stretchable material, which can be made into certain shapes through hot-pressing technique. One of the main problems of polyethylene membranes is there will be indentations during manufacture process. This will cause axial non-uniformity of the material and affects actuator's motion. The other one is cylindrical latex membrane, being widely used in soil sample analysis. We prepare different specifications of membranes (different diameters, thickness, etc.) for actuator in order to fulfill design and test requirements.

Latex is a kind of hyperelastic, incompressible material. In small-strain areas, classic elasticity theory can well explain the strain–stress relation within the elastic material. However, latex membranes will have large strain under external force. To solve the large deformation (finite length deformation) issues of this kind of polymer, the strain energy function should be introduced. There are three commonly used forms of function as follows: the Ogden function, the Mooney–Rivlin function, and the neo-Hookean function (Mooney, 1940; Gent, 1996; Ogden, 1997). Here, we choose the Ogden strain energy theory. The three-form of the Ogden function is adopted as:

(1) $$\begin{array}{l} W\left(\lambda_{1},\lambda_{2}\right)=\mu \overset{3}{\underset{i=1}{\sum }}\frac{\beta_{i}}{\alpha_{i}}\left(\lambda_{1}^{\alpha_{i}}+\lambda_{2}^{\alpha_{i}}+\lambda_{1}^{-\alpha_{i}}\lambda_{2}^{-\alpha_{i}}-3\right) \end{array}$$

where μ, α_*i*_, and β_*i*_ are the inherent material constants. λ_*i*_ (*i* = 1, 2, 3) are strains in three orthotropic directions. We divide the latex cylindrical membrane model into three directions: the meridional direction, circumferential direction, and vertical direction, as shown in **Figure 3A**.

### 3.2. Analysis of Static Equilibrium

In this section, the static model will be established to analyze the relationship between the characteristic parameters of SPCAM, that is, the initial screw pitch *L*_0_, the screw pitch *L*_1_ after deformation, actuator's diameter *D*, active coil number of spring *N*_*a*_, thickness of the membrane *H*, pressure difference Δ*P*, and the output force *F*_*output*_. Latex is a kind of non-linear, material, so we adopt a finite element analysis model based on the Ogden strain energy theory. Since polyethylene film is non-stretchable, its approximate solution can be obtained by using simple geometrical methods combined with classical mechanics. The analysis is based on the following assumptions:

1. The membrane has axial symmetry and density uniformity.
2. Thickness of the membrane is much thinner than the size of the actuator.
3. The influence of lateral force is ignored.

When SPCAM is under the effect of pressure difference, the spring will be compressed by the flexible membrane. However, the spring has a helix angle, so the compressing force does not apply entirely in the axial direction. Considering the screw pitch *L*_1_ is small compared to the spring's diameter *D*, we assume that the force interference caused by helix angle has little influence on the Hooke's coefficient of spring, yet it still affects the force equilibrium of actuator, manifested as torque τ.

**Figure 2:**
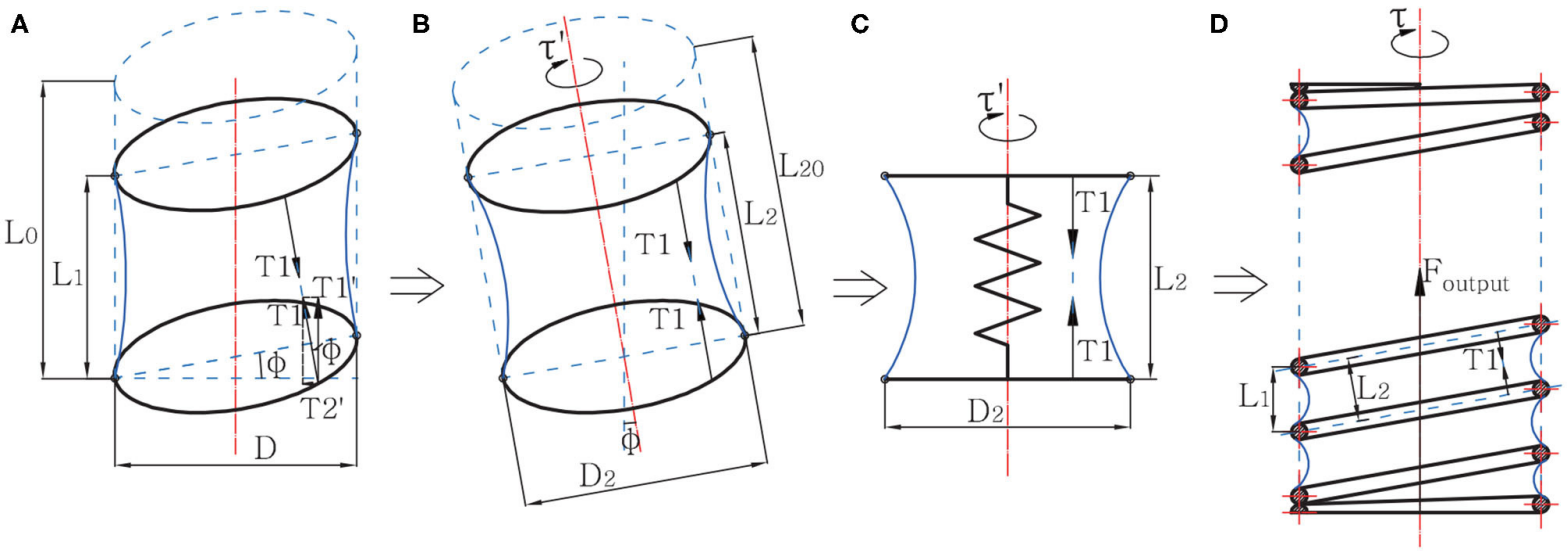
The equivalence process of the Self-sensing Pneumatic Compressing Artificial Muscle (SPCAM) simplified mechanical model. The dashed lines stand for a single helix part of SPCAM before deformation, while the solid lines stand for its state after deformation. **(A)** The original compression model of a single helix section under negative pressure. **(B)** The equivalent slant cylinder model of the single section. **(C)** The spring-cylinder model. Consider the helix as two parallel annuluses connected by an ideal spring, with the same Hooke's coefficient as the SPCAM's spring. **(D)** The mechanical model of the whole SPCAM, showing the output force *F*_*output*_ and torque τ.

The equivalence process of model analysis is displayed in Figure 2. On the spring helix, the effect of pressure on a membrane material particle generates a force *T*_1_, which has an included angle ϕ with the axis, where tan ϕ = *L*_1_/*D*. Therefore, a single helix in the actuator after deformation can be equivalent to a slant cylinder model shown in Figure 2B, where its height *L*_2_ = *L*_1_/ cos ϕ, and diameter *D*_2_ = *D*/ cos ϕ. Furthermore, considering the spring's compressive force, the model is eventually equivalent to a spring-cylinder model fixed at one end, as shown in Figure 2C. We can obtain the axial force *T*_1_ by analyzing this model. See model **a** in Figure 2. *T*_1_ can be divided into a SPCAM axial force $T_{1}^{\prime }=T_{1}\cos\phi$, and a radial force $T_{2}^{\prime }=T_{1}\sin\phi$. $T_{2}^{\prime }$ of all material particles has the same torsion direction, thus it creates a torque τ on the spring, making the spring twist for a certain angle. The final output force of the actuator, *F*_*output*_, can be expressed as follows:

(2) $$\begin{array}{l} F_{output}=F_{p}+T_{1}^{\prime }\pi D-K\Delta x \end{array}$$

where *K* and Δ*x* are stiffness coefficient and displacement (stretching or contracting) of the spring, respectively. *F*_*p*_ is the pushing force applied on the end of SPCAM by vacuum pressure,

(3) $$\begin{array}{l} F_{p}=\frac{\pi D^{2}}{4}\Delta P \end{array}$$

The interference torque τ is calculated as:

(4) $$\begin{array}{l} \tau =T_{2}^{\prime }\cdot \pi D\cdot \frac{D}{2}\cdot N_{a} \end{array}$$

Note that there might be relative sliding between the spring and the membrane, caused by τ. For different kinds of materials, the friction may not be the same, which is beyond the scope of this article.

Contraction rate is an important parameter of linear actuators. The contraction rate of SPCAM is related to the initial screw pitch *L*_0_ and membrane thickness *H*. If the change of membrane thickness is ignored during deformation process, the largest contraction strain of SPCAM can be approximately written as:

(5) $$\begin{array}{l} \delta_{contraction}=\frac{N_{a}\left(L_{0}-2H\right)}{L_{SPCAM}} \end{array}$$

where *L*_*SPCAM*_ is the total length of SPCAM.

Polyethylene film is non-stretchable, so only latex-membrane SPCAMs have the issue of stretching rate. The stretching strain is mainly limited by the fixing and clamping force on both ends of the latex membrane, and the breaking strength of latex material itself. The maximum stretching length Δ*X*_*stretching*_ satisfies the following constraints:

(6) $$\begin{array}{l} \{\begin{array}{l} T_{1}^{\prime }\left(\Delta X_{stretching},\Delta P\right)\pi D<F_{fixed}\\ \frac{T_{1}^{\prime }\left(\Delta X_{stretching},\Delta P\right)}{H}<\sigma_{latex} \end{array} \end{array}$$

#### 3.2.1. Latex SPCAM Model

From the equivalent model mentioned above, we can conclude that we only have to analyze the spring-cylinder model in Figure 2C to get the solution of *T*_1_, and eventually obtain the output force *F*_*output*_ and interference torque τ expressed in Equations (2–4). The static equilibrium of cylindrical hyperelastic materials can be seen in Guo (2001) and Soleimani and Funnell (2016). Different from those literature, our actuator only has one fixed end, and the other end will have axial compressing displacement, which makes the modeling process pretty troublesome. To simplify the model, we assume that the actuator will first come to an intellectual state. In this process, the actuator is not under any external force (or is under an infinitesimal force), but has the meridional uniform deformation, reaching a contracting (or stretching) state. That is, λ_1_(*X*) ≡ *L*_20_/*L*_2_ = λ_0_, where *L*_20_ is the height of slant cylinder model before deformation, and *L*_2_ is the distance between the two annuluses after the deformation. Then from this intellectual state, we fix the other end and analyze the compression deformation. The cylindrical membrane has the initial radius of midsurface *R*_*m*_, mounting length *L*_2_, and thickness *H*_*m*_. The undeformed membrane is referred to a cylindrical polar coordinate system (*X*, ϕ, *R*), while the deformed membrane is referred to a different cylindrical polar coordinate system (*x*, ϕ, *r*). The material particle moves from its position in the undeformed profile C(*X*, ϕ, *R*) to a new position in the deformed profile c(*x*, ϕ, *r*). For each particle, we have defined the principal stretches in the meridional λ_1_, the circumferential directions λ_2_, and the direction λ_3_ normal to the deformed membrane surface as:

(7) $$\begin{array}{l} \lambda_{1}=\frac{\text{d}s}{\text{d}S},\lambda_{2}=\frac{r}{R_{m}},\lambda_{3}=\frac{h}{H_{m}}=\frac{1}{\lambda_{1}\lambda_{2}} \end{array}$$

where s is the arc length measured from the pole (*x* = 0) to the particle c(*x*, ϕ, *r*) along the meridian of the deformed profile; S is the length measured from the pole (*X* = 0) to the particle C(*x*, ϕ, *R*) in the undeformed profile, where *S* ≡ *X*. *h* is the thickness of the membrane in the deformed situation, and λ_3_ is determined by assuming that the membrane is incompressible. From the Ogden function, intellectual state transformation and the analysis in Guo (2001), λ_1_, λ_2_, and θ can be expressed as:

(8) $$\begin{array}{l} \frac{\text{d}\lambda_{1}}{\text{d}x}=\lambda_{0}\cdot \frac{\sin\theta }{R}{\left(\frac{\partial^{2}W}{\partial \lambda_{1}^{2}}\right)}^{-1}\left(\frac{\partial^{2}W}{\partial \lambda_{1}\partial \lambda_{2}}\cdot \lambda_{1}-\frac{\partial W}{\partial \lambda_{2}}\right) \end{array}$$

(9) $$\begin{array}{l} \frac{\text{d}\lambda_{2}}{\text{d}x}=\lambda_{1}R^{-1}\sin\theta \end{array}$$

(10) $$\begin{array}{l} \frac{\text{d}\theta }{\text{d}x}={\left(\frac{\partial W}{\partial \lambda_{1}}\right)}^{-1}\left(\frac{\lambda_{1}\lambda_{2}}{H}\cdot \Delta P-\frac{\cos\theta }{R}\cdot \frac{\partial W}{\partial \lambda_{2}}\right) \end{array}$$

The geometric constraints at the boundaries are specified as follows:

(11) $$\begin{array}{l} x{|}_{X=0}=0,\theta {|}_{X=0}=0,x{|}_{X=L_{2}/2}=\frac{L_{2}}{2},\lambda_{2}{|}_{X=L_{2}/2}=1 \end{array}$$

To get the numerical solution at the boundaries, we use the fourth-order Runge–Kutta method to run iterations on λ_1_, λ_2_, and θ. The visual results are also displayed in Supplementary Video using ABAQUS.

In this analysis, we mainly focus on the traction transmitted in the meridional direction of the material particle, noted as *F*_1_. And *F*_1_ can be expressed as:

(12) $$\begin{array}{l} F_{1}=h\cdot \lambda_{1}\cdot \frac{\partial W}{\partial \lambda_{1}}{|}_{X=L_{2}/2} \end{array}$$

After getting the exact λ_1_, λ_2_, and θ at the boundaries from the iteration, we can plug them into Equation (12) to get *F*_1_. However, this *F*_1_ has a included angle θ with the vertical direction axis of slant cylinder model. We need to transform *F*_1_ into *T*_1_, which can be calculated as:

(13) $$\begin{array}{l} T1=F1\cdot \cos\left(\theta \right){|}_{X=L_{2}/2} \end{array}$$

According to Equations (12)–(13), we can eventually get the output force and interference torque of the actuator.

#### 3.2.2. Polyethylene Film SPCAM Model

**Figure 3:**
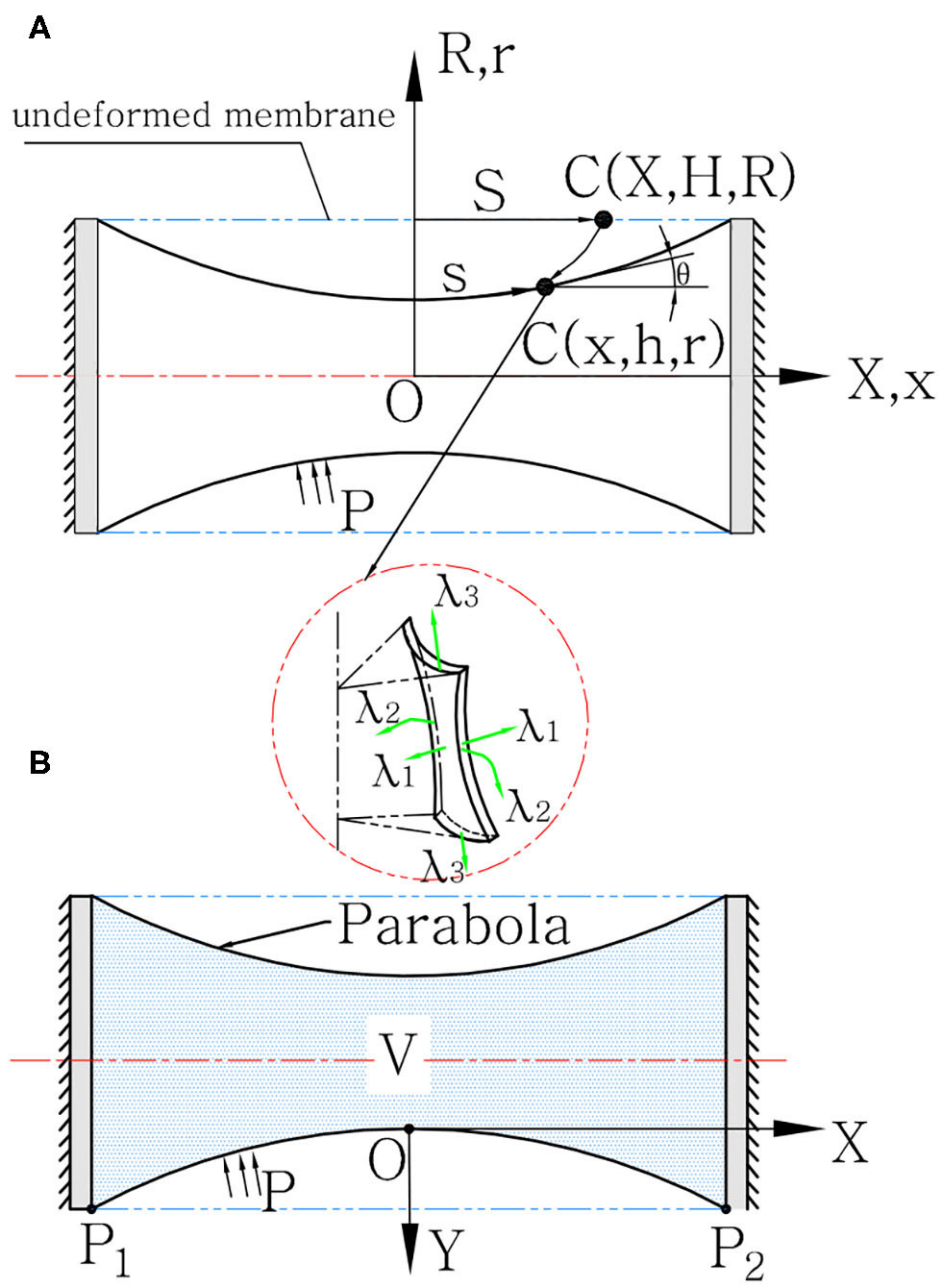
SPCAM's quasi-static model. **(A)** The profiles of undeformed and deformed hyperelastic latex membrane under coordinate systems (X, H, R) and (x, h, r), respectively. The detail view illustrates the principle stretches λ_1_, λ_2_, and λ_3_ of a material particle. **(B)** The profile of deformed polyethylene membrane under the coordinate system (x, y).

Same as latex membrane, a quasi-static cylindrical polyethylene membrane model is established to get *T*_1_. Here, the principle of virtual work and geometric approximation conditions are used to obtain the approximate solutions. Output forces have direct relations with vacuum pressure Δ*P* and model length *X* = *L*_2_, which together determine the curve of membrane. According to the principle of virtual work, resultant force *F* can be written as:

(14) $$\begin{array}{l} F\left(X\right)=T_{1}\left(X\right)\cdot \pi \cdot D_{2} \end{array}$$

(15) $$\begin{array}{l} F\left(X\right)\cdot \delta X=-\Delta P\cdot \delta V\left(X\right) \end{array}$$

where *V* represents the internal air volume. We built the coordinate system *XOY*, where *O* is on the center profile of the membrane (i.e., the point with maximum cavity), whose height is marked as *h*_*O*_. Two endpoints are marked as *P*_1_,*P*_2_, as shown in Figure 3B. The function of the parabola can be expressed as:

(16) $$\begin{array}{l} y=\frac{4h_{O}}{X^{2}}x^{2} \end{array}$$

Here, we approximately consider that the arc length of the parabola $\overset{⌢}{P_{1}OP_{2}}$ equals to twice the length of segment $\bar{P_{1}O}$. While the polyethylene material is non-stretchable, the length of membrane is constant, yielding:

(17) $$\begin{array}{l} \sqrt{X^{2}+h_{O}^{2}}=\frac{L_{20}}{2} \end{array}$$

The total volume *V* can be written as:

(18) $$\begin{array}{l} V\left(X\right)=2\cdot \int_{0}^{\frac{X}{2}}\pi {\left(\frac{4h_{O}}{X^{2}}l^{2}+\frac{D_{2}}{2}-h_{O}\right)}^{2}dl \end{array}$$

Combining the Equations (14, 15, 17, and 18), one achieves:

(19) $$\begin{array}{c} T_{1}\left(X\right)=\frac{-\Delta P}{D_{2}}(\frac{D_{2}^{2}}{4}-\frac{D_{2}\sqrt{L_{20}^{2}-X^{2}}}{3}-\frac{2X^{2}}{5}\\ +\frac{2L_{20}^{2}}{15}+\frac{D_{2}X^{2}}{3\sqrt{L_{20}^{2}-X^{2}}}) \end{array}$$

Note that when *X* = *L*_20_, *T*_1_ tends to be infinity. Theoretically, because of the membrane's non-stretchability, the actuator cannot be in the non-compression state under vacuum pressure. Meanwhile, the model is approximate, so there will be rather big errors under small deformation.

## 4. Evaluation Experiments

### 4.1. Experimental Setup

**Figure 4:**
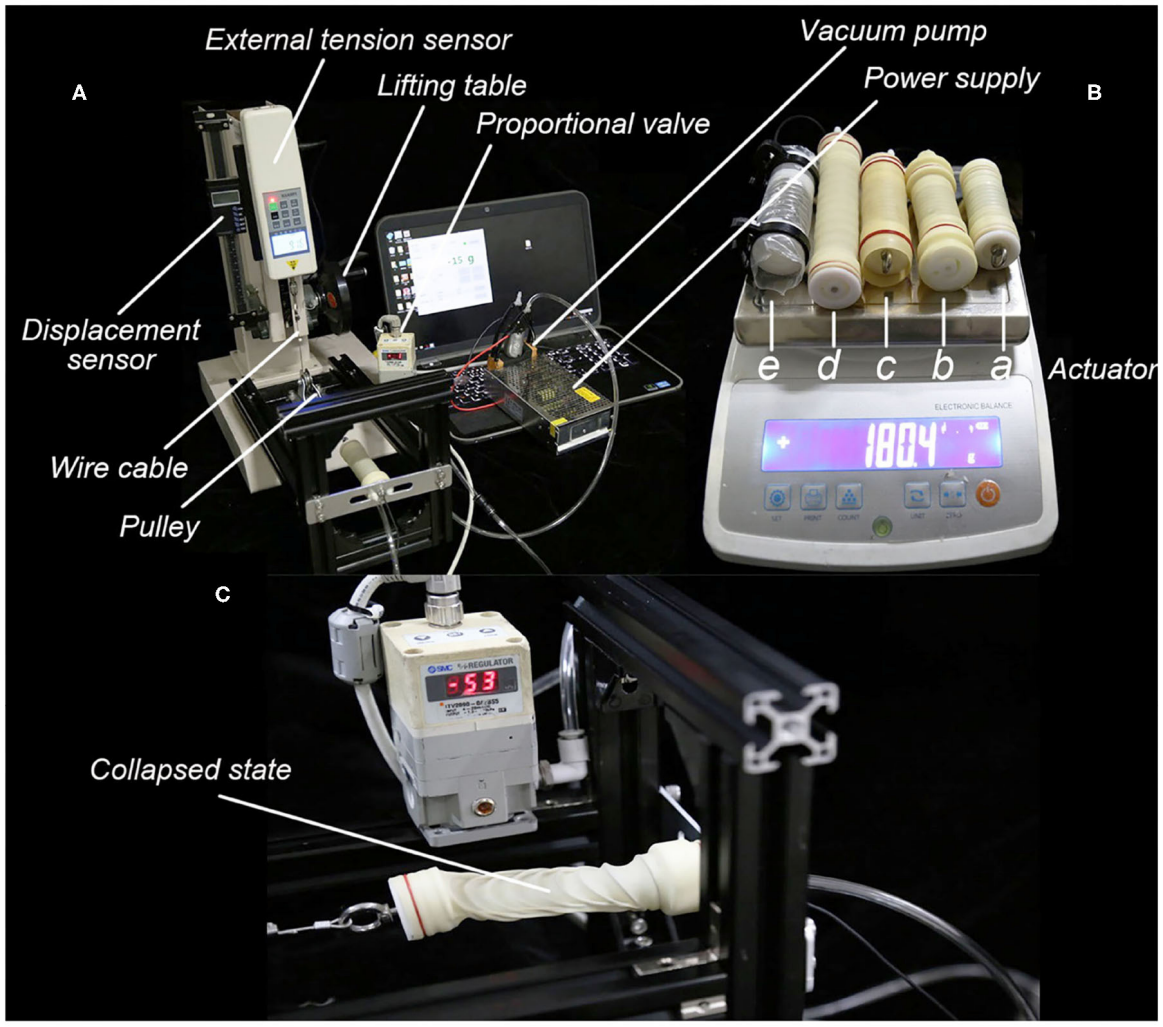
The experimental setup and actuators for test. **(A)** The platform built to test the characteristics of Self-sensing Pneumatic Compressing Artificial Muscle (SPCAM), with the displacement sensor and the external tension sensor. **(B)** Five different actuators to be tested, with the average weight <40 g. Actuator a, b, c, and d are made up of latex membrane with different thickness, while actuator e is made up of polyethylene film. Specific parameters are listed in Table 2. **(C)** The collapse phenomenon of latex membrane actuator. When overstretched and under high negative pressure, the actuator collapses sidewise due to the unbalanced force, and is unable to work normally.

A platform was built to test the static characteristics of SPCAM and evaluate the results of finite element analysis, as shown in Figure 4. The platform mainly comprises a lifting table, a capacitive displacement transducer, and an external tension sensor. One end of the actuator is fixed to the base, and the other end is connected to the external tension sensor via a pulley, thus we can detect the output force. We used the lifting table to adjust the displacement of the actuator, which would be recorded by the displacement sensor. A vacuum pneumatic proportional valve (SMC, ITV-2090-042BS5, –80 kPa max.) is connected to the miniature vacuum pump (kamoer, KVP08, –82 kPa max.) to control the internal pressure of actuator. Before the experiment, the Hooke's coefficient of spring was measured first. For the membrane material, we chose three kinds of latex membrane with the diameter of 25 mm and the thickness of 0.3, 0.5, 0.8 mm, respectively, and a kind of LDPE membrane with the thickness of 0.2 mm. Five actuators were made in total for test, whose parameters are listed in Table 2. We tested the displacement detection accuracy of the built-in sensor and the output force of SPCAM under static equilibrium (considering no effects of dynamic force) and compared it with simulation results. Meanwhile, we displayed the actuator's ability of interference detection and shock resistance.

**Table 2:** Parameters of five actuators.

**Actuator**	**Overall length (mm)**	**Active coil number**	**Hooke's coefficient (N/m)**	**Membrane thickness (mm)**	**Membrane material**
a	85	8.1	135.77	0.3	Latex
b	85	8.1	135.77	0.5	Latex
c	85	8.1	135.77	0.8	Latex
d	140	9.5	158.80	0.5	Latex
e	85	8.1	135.77	0.2	LDPE

### 4.2. Sensor Accuracy Test

**Figure 5:**
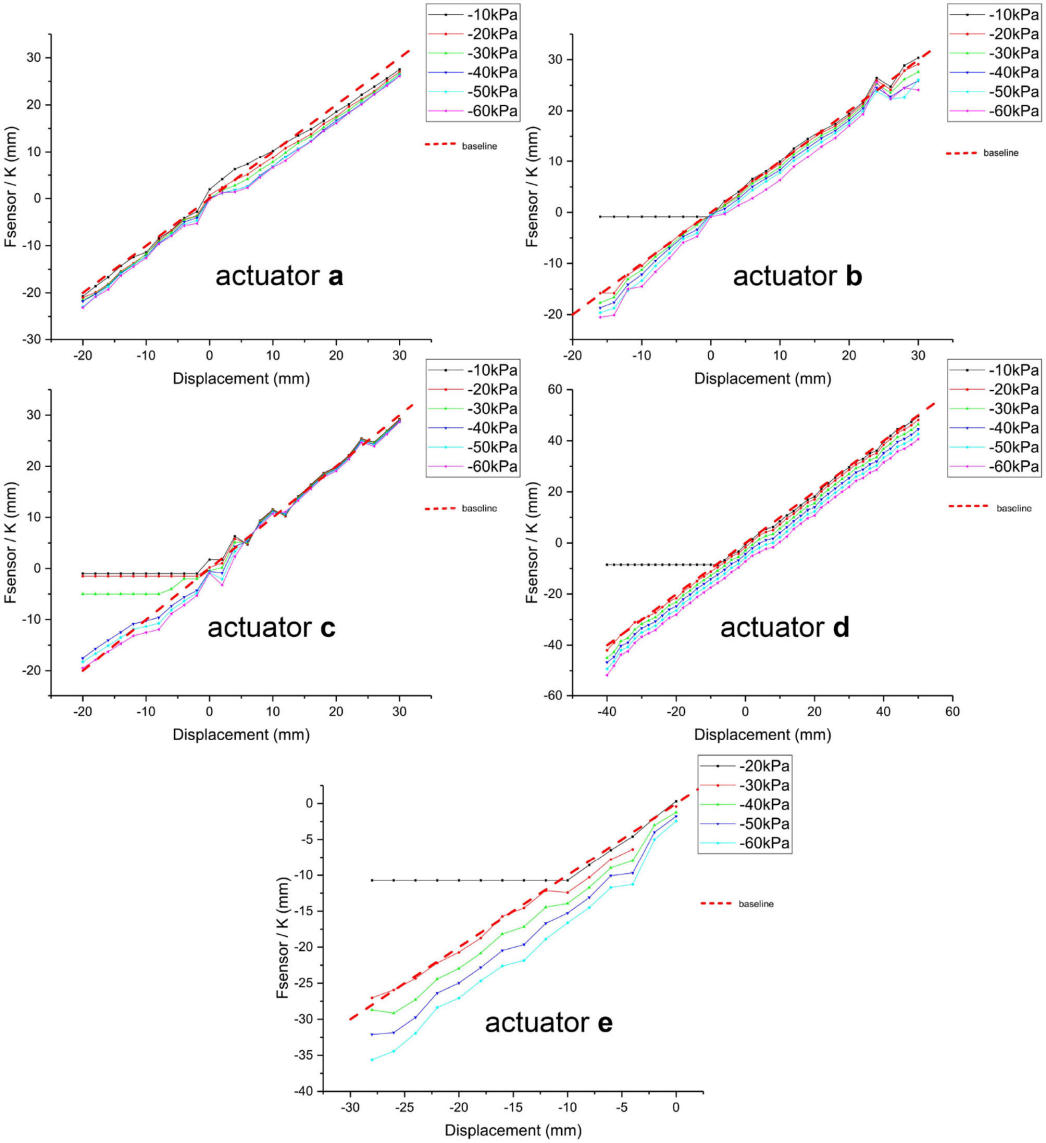
Accuracy of five actuators under quasi-static equilibrium. The displacement detection error of the sensor is represented by the distance between experimental data points and the reference line in the vertical direction. Negative value of the abscissa indicates that the actuator is in contraction state, while positive value means stretching state. Horizontal experimental data line indicates that the actuation force is too weak to actuate the SPCAM, leading to the relaxed state.

Theoretically, the output force of the built-in microsensor should depend linearly on the actuator's displacement, with the scale factor of *K* (i.e., the Hooke's coefficient of the spring). During the experiment, we applied different pressure to the actuator at each displacement point in the static equilibrium state, and sensor's measured value is expected to be constant regardless of the change of vacuum pressure. Thus, the fluctuation of the measured value caused by pressure change is the main motion error of SPCAM. Figure 5 shows the relations between detected and actual displacements of five actuators, respectively. Different colors of solid lines represent different pressures, varying from 10 to 60 kPa. The red dashed line with the slope of 45° presents the reference value. The abscissa presents the actual displacement of the steel wire, which is connected to the end of the actuator, so it is also the absolute displacement of the actuator when the wire is tensioned. Its value is measured by a high-precision capacitive sensor. Negative value of the abscissa indicates that the actuator is in contraction state, while positive value means stretching state. The ordinates is the measured value *F*_*sensor*_ of built-in sensor divided by *K*. The position error is displayed as the height difference to the base line vertically. For actuators a–c, the maximum position errors are 4.0, 6.1, and 5.2 mm, and root-mean-square errors (RMSEs) are 2.2, 1.8, and 1.3 mm, respectively (erroneous data are excluded). For actuator d with the total length of 140 mm and maximum displacement about 100 mm, the RMSE is 4.5 mm, therefore the relative displacement error is <5%. The error tends to increase with the increase of pressure. This is mainly because the spring twists along with the contracting (or stretching) process, which changes the spring's structure, thus affects the Hooke's coefficient. Besides, the contraction force *T*_1_ of the latex membrane does not uniformly distribute on the spring due to installation error, which results in an additional lateral force and further increases the error.

Meanwhile, when the negative actuating pressure is too low and the contraction displacement exceeds a certain limit, the actuator will come to a relaxed state and is unable to work normally, like the black curves representing –10 kPa pressure of actuators b–e in Figure 5. And this is also directly related to the thickness of the membranes. Negative pressures must apply work on the membrane, so the thicker the membrane, the more energy is needed to deform it, and the more obvious the relaxed phenomenon.

As for different membrane materials, the sensor error of actuator e (maximum position error 8.4 mm, and RMSE 3.4 mm) using polyethylene membrane is obviously larger than that of latex membrane actuators. This is because polyethylene membrane's manufacture process creates hot pressing indentations, which causes its inherent axial non-uniformity, and its inextensibility with incompressibility worsens the situation.

In addition, we observed that if SPCAM with latex membrane was in the stretching state, beyond certain displacement, it would collapse sidewise (Figure 4C) due to unbalanced forces (disturbing force and lateral force) when vacuum pressure reached a critical point, similar to buckling deformation of springs. To avoid this situation, the maximum tensile strain of SPCAM should be carefully limited.

### 4.3. Output Force Test

**Figure 6:**
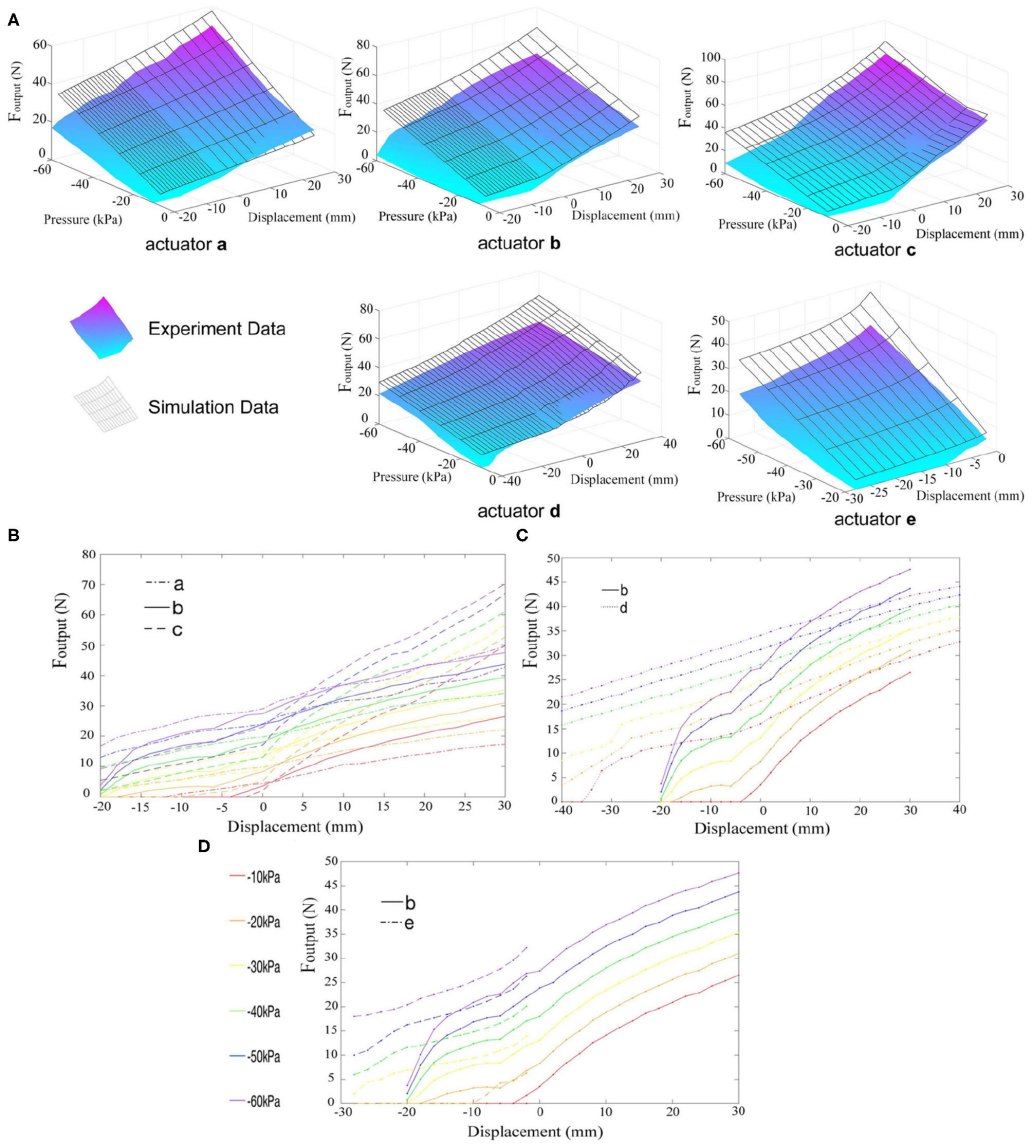
The relations between output force and actuation pressure, displacement of the actuators. **(A)** The simulation results and experiment data of five different actuators. The black surface represents the results of simulation as described above, while the colored surface represents the experimental results. **(B–D)** The comparison of output forces of actuators with different parameters. Different colors represent different actuation pressure, varying from –10 to –60 kPa. Actuators a–c in **(B)** have different membrane thickness (0.3 mm for actuator a, 0.5 mm for actuator b, and 0.8 mm for actuator c). Actuators b and d in **(C)** have different length, and the longer actuator d has larger strain. In **(D)**, actuator b is made up of latex membrane, while actuator e is made up of polyethylene film.

The actuator can produce output force more than 50 times larger than its weight. The relationship among pressure, displacement, and output force was investigated in output force test, and the result is shown in Figure 6. The black surface represents the results of simulation as described above, and the colored surface represents the results of experiments. We can see that the output force obviously increases when vacuum pressure increases. When the spring is in compressing state, output force gradually decreases if displacement increases. On the contrary, when the spring (the latex membrane actuator) is in stretching state, output force increases with the increase of displacement, which resembles animal muscles' characteristics.

Comparing experiment results with finite element analysis, overall trend is the same, but there are still certain differences. Almost all numerals of simulation results are larger than experiment results. This may because some simplifying assumptions used in finite element analysis do not completely conform to actual conditions. For example, the torque τ has severe influence on long actuator d, making the spring twist too much, enlarging the lateral friction between spring and membrane, and largely decreasing the output force. Further analysis will be made to model the friction accurately. At present, under the condition of low output force, the simulation results do not deviate much from the experimental ones.

Besides, the resultant force of the membrane is not completely in the axial direction, which also makes most experiment results generally smaller. As shown in Figure 6B, as the thickness of the membrane becomes larger, the output force of the actuator also increases substantially. Because the surface tension of the latex membrane is positively correlated with the membrane thickness, if the membrane is too thin, it cannot resist vacuum force, causing a severe inward deformation and a large angle θ (shown in Figure 3A) between the force and the axial, thus reduces the output force (actuator a). However, if the membrane is too thick, it is disadvantageous for low-pressure actuation. As the aforementioned relaxated state phenomenon, when the latex membrane is too thick, the vacuum force is insufficient to deform it. Therefore, actuator c with the membrane thickness of 0.8 mm has almost zero output force under negative pressure of 0–20 kPa when it is in the contraction state. The length of the actuator and the initial screw pitch of the spring also affect the actuator's performance (Figure 6C). Long actuators have more effective coils, thus have larger strain. Under the same pressure, with the same contraction displacement or low stretching displacement, output force of actuator d is obviously larger than that of actuator b due to relaxed state phenomenon. But when the stretching displacement is beyond 25 mm, latex membrane of actuator b has larger axial deformation rate. In this case, the tension in the membrane becomes the dominant factor, which makes the output force of actuator b slightly larger than that of actuator d. As a result, long actuator has better performance than short one concerning total output force and maximum displacement. However, actuator d has larger error under the same displacement due to the influence of the torque τ. At the same time, its stability becomes markedly lower and is more likely to collapse.

Although the non-stretchability of polyethylene membrane on actuator e can help enlarge output force, the improvement is not so significant compared to latex membrane, shown in Figure 6D. This is because the extensibility of latex makes it possible for the membrane to totally fit on the spring under initial configuration. polyethylene membrane, however, is non-stretchable, so its inner diameter should be a little larger than spring's diameter when installed, which affects output force to some extent. Meanwhile, polyethylene membrane actuators cannot work in the stretching state, which severely impacts actuator's stretching rate. So we prefer to choose latex membrane rather than polyethylene film used in FOAMs (Li et al., 2017) under light load.

### 4.4. Dynamic Response and Impact Test

**Figure 7:**
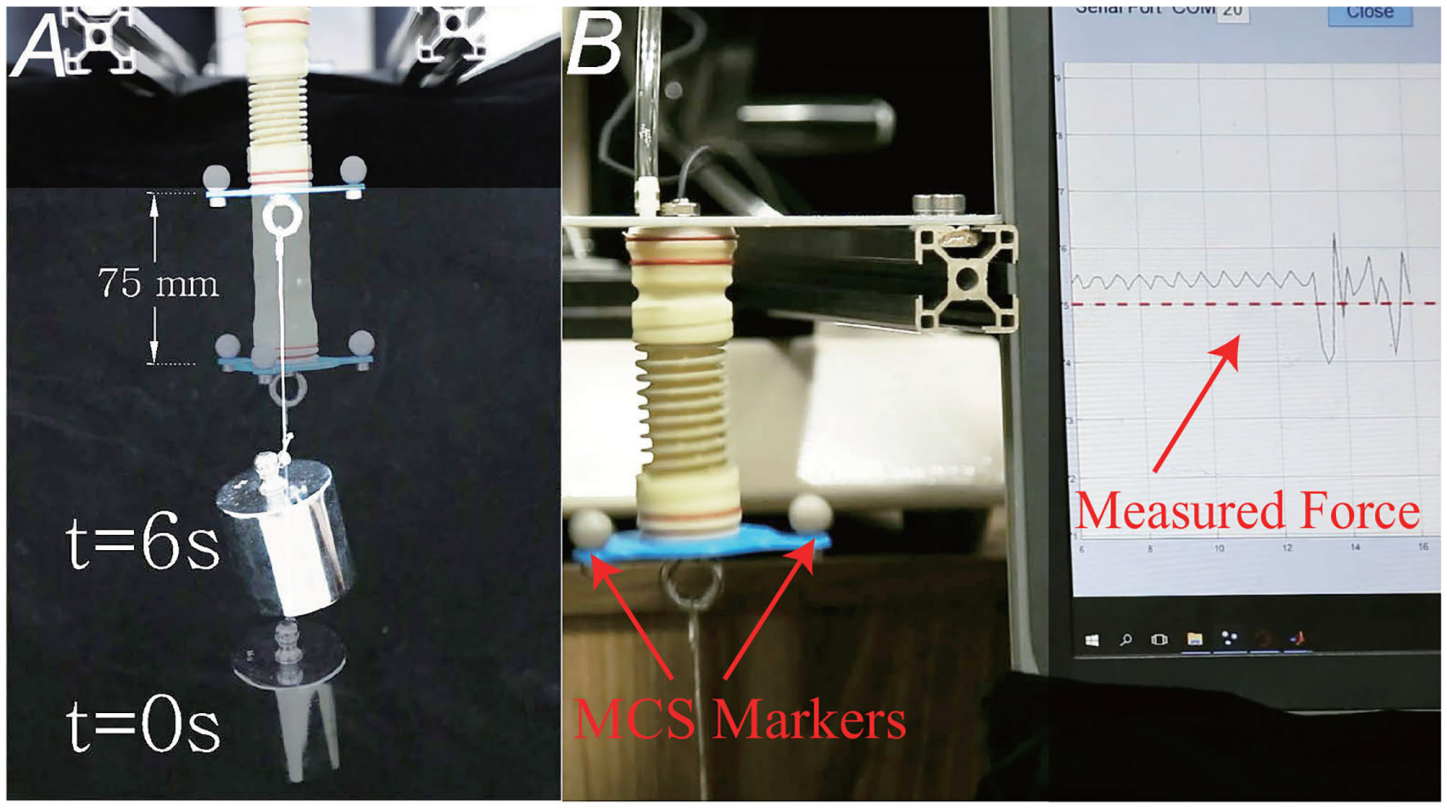
Demonstration of the dynamic performance and impact tests. **(A)** The position before and after actuation. The actuator is able to lift the 1 kg weight for 75 mm within 6 s. **(B)** The actuator is able to withstand shocks, as well as roughly detect the intensity of the impact, and whether it touches the target object or interferes with obstacles. When the external impact happens, the internal sensor's measured value will have a large fluctuation.

We roughly tested the actuator's dynamic performances. A motion capture system (OptiTrack, Prime 17Wx12, 0.5 mm location accuracy) was used to detect the actuator's displacement. Actuator *d* was chosen for the test, with a 1 kg weight on the end. A feedback control algorithm was adopted to control the actuators' position. Results are shown in Figure 7A. The actuator is able to lift the 1 kg weight for 75 mm within 6 seconds, only with feedback of the built-in sensor. The motion error is about 2.5 mm, measured by motion capture system. Furthermore, the actuator's dynamic response speed depends on the flow rate of the air pump and proportional valve. If we use air pump with larger flow, the speed of movement can be further increased.

One of the major advantages of the soft actuator is its inherent flexibility. Since the actuator comprises elastic parts like latex membrane and spring, similar to series elastic actuators (Pratt and Williamson, 1995), it can resist external impacts and guarantee safety. Not only can the actuator withstand shocks, it is also able to roughly detect the intensity of the impact, and whether it touches the target object or interferes with obstacles, as illustrated in Figure 7B. When the external impact happens, the internal sensor's measured value will have a large fluctuation, then the detector is triggered. Besides, the impact strength can be considered to be proportional to the fluctuating value.

## 5. Positive-Negative Pressure Combined Actuation

Another core advantage of SPCAM is that it can combine negative and positive pressure actuation. Output force of previous SPCAM or FOAMs is limited by maximum negative pressure (–100 kPa max.), while positive pressure can reach a large value. Another issue is when actuating pressure rises, requirements for pressure sources become stricter, which leads to clumsy air pumps or compressors, accompanied by huge noises. Meanwhile, high pressure actuation also brings in potential safety threats. This is undesirable for most mobile robots and wearable devices.

To solve these problems, the method of combined actuation for soft actuator has been first proposed. When there is already negative pressure within SPCAM, we can provide positive pressure outside the membrane for actuation. Obviously, under low-pressure actuating circumstances, positive-negative pressure combined actuation has much lower requirements on devices than negative or positive pressure actuation alone. For example, using an equipment combining 50 kPa positive pressure with –50 kPa negative pressure will have much lower weight, noise, and cost than an air pump or vacuum pump that generates 100 kPa pressure difference. Based on such principles, we designed two different kinds of positive-negative pressure combined actuation structures. This can improve the actuator's loading ability and safety, which greatly broadens the application prospect of the actuator in low-pressure actuating field.

### 5.1. Lightweight Air Cylinder

**Figure 8:**
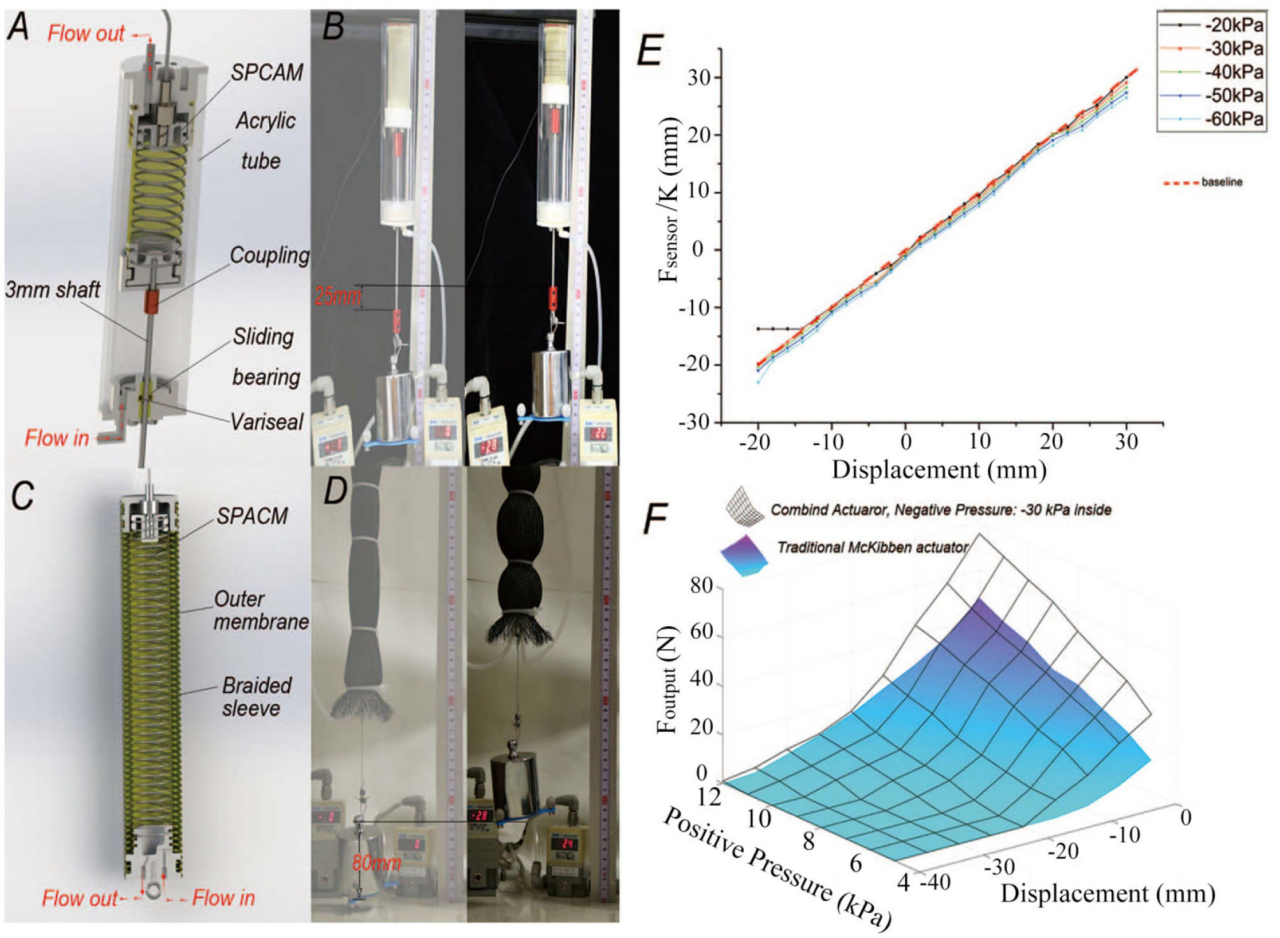
The structures and performance of two different types of combined actuator. **(A)** The schematic of negative–positive pressure combined air cylinder. **(B)** Demonstration of the combined air cylinder actuator. The actuator can still get satisfactory output force and displacement even both positive and negative pressure difference is under 30 kPa. **(C)** The structural drawing of the second type of actuator which combined the SPCAM with McKibben artificial muscle. **(D)** The actual performance of the second combined actuator. It is able to lift the 1 kg weight for about 80 mm. **(E)** The accuracy of air cylinder combined actuator. The RMSE of the combined actuator is about 1.0 mm and the maximum error is 3.5 mm, much smaller than that of original actuator b alone. **(F)** The relation among displacement, actuation pressure, and output force of McKibben-combined actuator and traditional one. Under low-pressure actuation, the output force of the combined actuator (represented by the black surface, -30 kPa vacuum pressure inside) is larger than that of the traditional artificial muscle (colored surface) and maximum contracting displacement also increases.

First, we designed an actuator combining a traditional air cylinder, whose structure is shown in Figure 8A. The wall of the air cylinder is a 180 mm long and 5 mm thick acrylic tube with SPCAM actuator **b** inside of it. A precision-machined shaft with the diameter of 3 mm is connected to the end of SPCAM and a sliding seal structure is adopted at the end face of the acrylic tube. Two sliding bearings are used to constrain shaft's degree of freedom in the radial direction, and a micro variseal is used to seal high-pressure air. By applying positive pressure to the acrylic tube and negative pressure to SPCAM, we can produce larger pressure difference or lower the requirements for air source equipments. Figure 8B shows that the actuator can still generate satisfactory output force even both positive and negative pressure difference is under 30 kPa. Meanwhile, due to the effects of sliding bearings, the influence of lateral force reduces sharply and position accuracy of SPCAM is further improved, as can be seen in Figure 8E. The RMSE of combined actuator is about 1.0 mm and the maximum error is 3.5 mm, much smaller than that of actuator b.

### 5.2. McKibben Artificial Muscle

McKibben artificial muscle is a widely used pneumatic actuator. It is usually considered to be biological muscle like for its similarity in real muscle contraction and relaxation. Besides, the basic working mechanism endows itself with great variable compliance dependent on applied pressure. But one of its problems is the need for higher air pressure. A lot of literatures have researched the property of McKibben artificial muscle and tried to improve its performance. Here, we designed a combined actuator having SPCAM put inside the McKibben artificial muscle, as shown in Figures 8C,D. The outer part of the combined actuator consists of latex membrane and nylon shell with the diameter of 38 mm, similar to the McKibben artificial muscle in Chou and Hannaford (1996). The internal part is our SPCAM. When applying positive pressure Δ*P*_1_ to the McKibben muscle and negative pressure −Δ*P*_2_ to the SPCAM, the McKibben actuator shortens under positive pressure Δ*P*_1_, while SPCAM contracts under pressure difference Δ*P*_1_ + Δ*P*_2_, achieving larger output force and contraction.

The Model can be resolved into a SPCAM model and McKibben artificial muscle. The simplified artificial muscle model proposed in Chou and Hannaford (1996) is used to analyze the output force. For the McKibben artificial muscle, we can know from the virtual work principle:

(20) $$\begin{array}{l} dW_{in}=\Delta P_{1}\cdot dV_{McKibben}=dW_{out}=-F_{McKibben}\cdot dX \end{array}$$

where Δ*P*_1_ is the provided positive pressure, *dV*_*McKibben*_ is the volume change of the McKibben Artificial Muscle, and *dX* is its effective length.

(21) $$\begin{array}{l} F_{McKibben}=-\Delta P_{1}\frac{dV_{McKibben}}{dX} \end{array}$$

The model of braided shell has following geometric relationships:

(22) $$\begin{array}{l} X=b\sin\theta \end{array}$$

(23) $$\begin{array}{l} D_{McKibben}=\frac{b\sin\theta }{n\pi } \end{array}$$

where θ is the angle between a braided thread and the cylinder long axis, *D*_*McKibben*_ is the diameter of the McKibben actuator cylinder, n is number of turns of a thread, and b is the thread length, same as what has been discussed in Chou and Hannaford (1996).

Approximately consider the artificial muscle as a cylinder, and assume that the deformation of SPCAM does not affect the volume change of the air inside the McKibben artificial muscle, the volume of McKibben actuator can be expressed as:

(24) $$\begin{array}{l} V_{McKibben}^{\prime }=\frac{1}{4}\pi \left(D_{McKibben}^{2}-D_{SPCAM}^{2}\right)X\\ \;=\frac{b^{3}}{4\pi n^{2}}\sin^{2}\theta \cos\theta -\frac{\pi bD_{SPCAM}^{2}}{4}\cos\theta \end{array}$$

Combine Equations (21)–(23):

(25) $$\begin{array}{l} \begin{array}{c} F_{McKibben}^{\prime }=-\Delta P_{1}\frac{dV_{McKibben}^{\prime }}{dX}=-\Delta P_{1}\frac{dV_{McKibben}^{\prime }/d\theta }{dX/d\theta }\\ =\frac{\Delta P_{1}b^{2}\left(2\cos^{2}\theta -\sin^{2}\theta \right)}{4\pi n^{2}}+\frac{\Delta P_{1}\pi bD_{SPCAM}^{2}}{4}\sin\theta \\ =\frac{2\Delta P_{1}b^{2}-3\Delta PX^{2}}{4\pi n^{2}}+\frac{\Delta P_{1}\pi XD_{SPCAM}^{2}}{4} \end{array} \end{array}$$

Meanwhile, the internal SPCAM is under the pressure difference of Δ*P* = Δ*P*_1_ + Δ*P*_2_, and generates output force *F*_*SPCAM*_(Δ*p, b*sinθ), which can be obtained in modeling analysis section previously. The final output force *F*_*output*_ is:

(26) $$\begin{array}{l} F_{output}=F_{McKibben}^{\prime }+F_{SPCAM} \end{array}$$

While the output force of the original McKibben artificial muscle is:

(27) $$\begin{array}{l} F_{McKibben}=\frac{2\Delta P_{1}b^{2}-3\Delta PX^{2}}{4\pi n^{2}}<F_{output} \end{array}$$

After adopting the combined actuating method, the actuator has not only greater output force but also larger displacement than the traditional McKibben artificial muscle under the same maximum pressure. In the experiment, we tested the performance of combined actuator and traditional McKibben muscle. For the combined SPCAM, the negative pressure −Δ*P*_2_ here is simply set identically as –30 kPa. The relation among the actuator's displacement, actuating pressure, and output force is displayed in Figure 8F. It can be seen that under low-pressure actuation, the output force of the combined actuator is larger than that of the traditional artificial muscle and maximum contracting displacement also increases.

## 6. Application

In this section, we designed two prototypes to demonstrate the great advantages of SPCAM and positive–negative pressure combined actuation. The first one is a soft robot gripper, which can resist impacts and ensure safety. It can also detect impacts and grasping state, that is, whether it has grasped the object firmly. The second one is a robot joint using Mckibben-SPCAM actuator. Also it has the potential to be used on wearable devices or exoskeletons.

### 6.1. Dexterous Robot Hand

**Figure 9:**
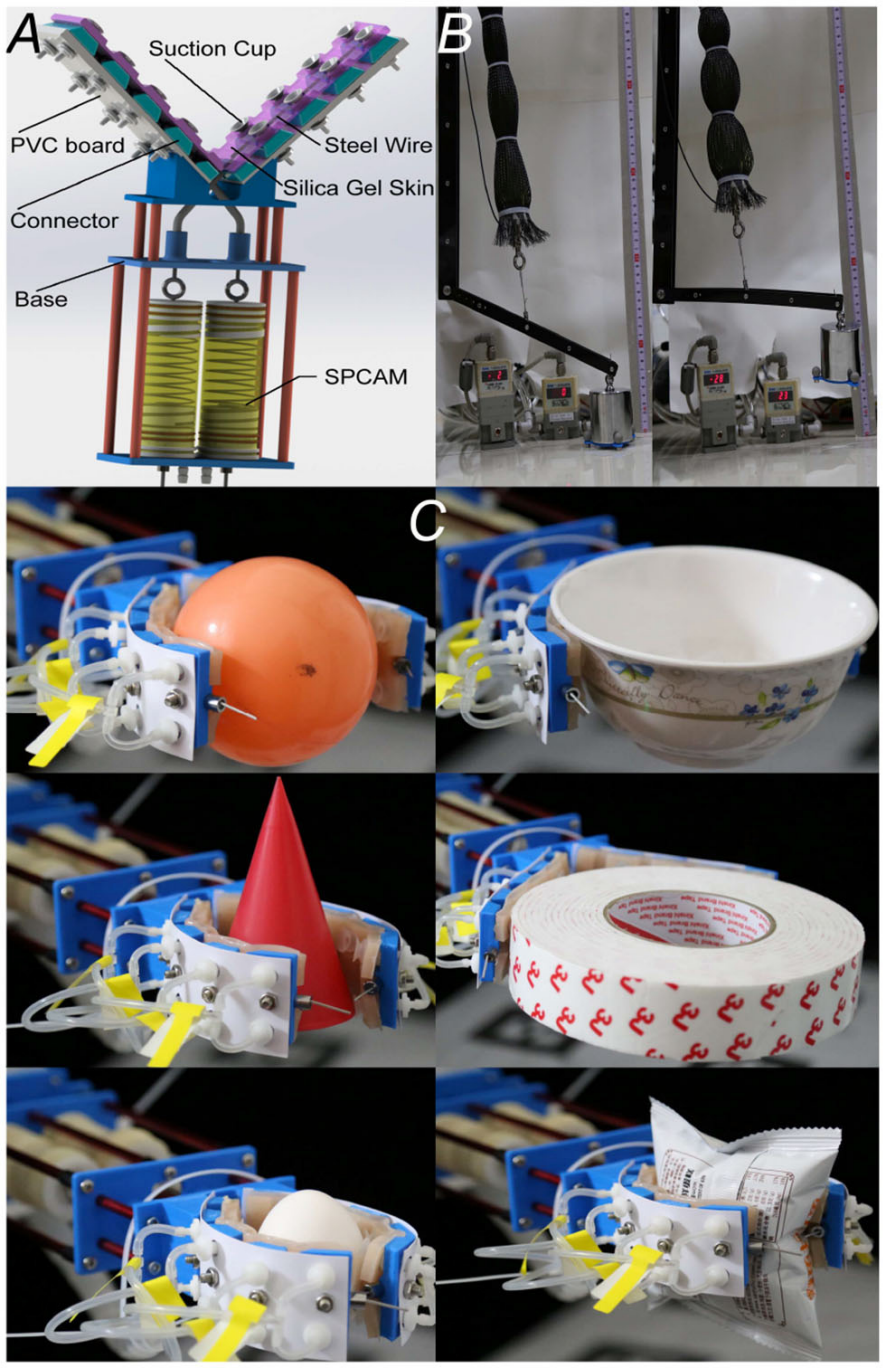
The applications of Self-sensing Pneumatic Compressing Artificial Muscle (SPCAM). **(A)** The structural schematic diagram of the two-finger dexterous robot hand. **(B)** The application of positive–negative pressure combined SPCAM on a robotic joint as exoskeleton equipment, resembling human's muscles. **(C)** Demonstration of the dexterous gripper when grasping different target objects, including fragile and soft ones. From left to right, top to bottom, are rubber ball, bowl, cone, tape, raw egg, and puffed food bag, respectively.

We designed a two-finger soft gripper with two degrees of freedom (DoFs), weighing about 240 g. The structure of the gripper is shown in Figure 9A. The gripper consists of two flexible fingers, tendon-driven mechanism, two SPCAM actuators, and base. On each finger, five 3D-printed hard connectors are used as phalanges. Suction cups are fixed on the phalanges, which share the same vacuum circuit with SPCAMs, which can generate suction force to help better grasp target objects. In order to increase friction force on contact surface, fingers are covered with silica gel skin. When the SPCAM contracts, the effective length of steel wire in the fingers shortens, and the elastic deformation of the PVC board happens between two neighboring 3D-printed connectors, causing the fingers bent and the gripper closed. Since the actuator is able to detect impacts and collisions, the gripper can sense its grasping state, so in an unstructured environment, it can be well adapted and ensure safety.

The robotic gripper is mounted on the end of a six DoFs manipulator (Kinova Company, *MICO*^2^). We first did experiments on impact resistance during grasping. Based on the detecting method mentioned before, we can find whether the gripper is influenced by impact loads readily. When fierce impacts occur, the actuator's structure still will not be damaged and is able to recover. Furthermore, using the information of force sensor to limit the output force, the gripper is capable of automatically grasping fragile or soft target objects like inflated packaging bags and raw eggs, without crushing them, which shows its dexterity and adaptivity (illustrated in Figure 9C).

### 6.2. Flexible Joint for Exoskeleton

Wearable devices and exoskeletons have been research hotspots in recent years. Here the SPCAM, combined with McKibben artificial muscle, is used on a robotic joint as exoskeleton equipment. The robotic joint has one rotation DoF, which connects the forearm with upper arm (both made up of acrylic board). The two ends of the combined actuator are fixed on the forearm and upper arm, respectively. When the actuator contracts, the forearm is pulled to rotate about the axis of joint, resembling human's muscles (Figure 9B). Besides, since the joint is flexible, it also has the ability to sense impacts. In the demonstration, the endpoint of forearm is loaded with a 1 kg weight, and it is able to lift the weight up to 70 mm.

This combined mechanism is suitable for exoskeleton actuator. The wearable devices and exoskeleton need to be lightweight, adaptive, quiet, and energy-saving, which can be well fulfilled by our positive–negative pressure combined actuators. In contrast, other actuators like traditional electric motor cannot guarantee the compliance to users, or they are heavy and costly.

A simple prototype is designed for demonstration. We choose two micro air pumps (ZQ370-03PM, positive pressure 80 kPa max., negative pressure –45 kPa max.). The actuator is able to generate maximum traction about 70 N, which can offer assistance to some extent. The weight of the whole equipments is <500 g, and the working noise is only 52 dB, which accords with the requirements of living environment.

## 7. Conclusion and Future Work

Soft actuator is the core technique in soft robot field. In this paper, we proposed a pneumatic actuator SPCAM, which realizes axial contracting movement. Our design uniquely uses stretchable latex as the membrane material for the actuator, bringing it higher strain rate, flexibility, and ability to resist impacts. The actuator has built-in microtension sensors, making it able to detect its absolute displacement and roughly sense the surrounding environment, like collisions and impacts. We established simplified mechanical models under quasi-static equilibrium for latex membrane and polyethylene film, respectively. Finite element analysis has been chosen to build the model for latex membrane due to the material's hyperelasticity, and for polyethylene film, we combined the virtue work principle with geometry approximation. Finally, the output force and interference torque are related with the actuation pressure and other inherent parameters of the actuator. This can help analyze the actuator's operating principle and system error, thus help optimize the design. Five actuators with different parameters are used for experiments, whose result are roughly identical with the simulation results. Latex membrane actuators are obviously better than polyethylene film actuators concerning overall performance, and the performance of actuators with different membrane thickness varies distinctly under different pressures. For example, the actuator with thicker latex membranes has larger output force under high pressure, but works unsatisfyingly under low pressure, while thinner membrane actuator has just the opposite performances. Meanwhile, the length of actuator has significant influence on the maximum displacement and torque. These theoretical and experimental results can guide us to choose proper actuators for different target applications in order to gain the best control effect.

Another essential highlight is the proposal of positive–negative pressure combined actuation, which can effectively reduce the demand of maximum actuation pressure. In the experiments, positive and negative pressure difference both no larger than 30 kPa can reach the same control effect as the normal SPCAM under larger pressure. This also guarantees safety during application like human–robot interaction, and lowers the requirements for air source equipments.

The SPCAM actuator still has some shortcomings. There are certain differences between simulation results and experiment data, mainly because too many simplification hypotheses were used in mechanical analysis, which does not completely accord to reality. A more precise model has to be built in the future in order to further optimize actuator's parameters. Data from pressure sensors and tension sensors also can be combined more effectively. For instance, we can compensate the force interference caused by vacuum pressure by detecting the actuator's internal pressure, thus obtaining higher detecting accuracy of displacement. Besides, if air source equipment with large flow is used, the actuator's response speed will be largely increased. In this case, the actuator's dynamic performance should be taken into account for it has considerable influence on precision, output force, and stability. Furthermore, in order to verify the actuator's reliability, further fatigue-limit tests should be done.

## Data Availability Statement

All datasets presented in this study are included in the article/Supplementary Material.

## Author Contributions

NL and HZ has contributed equally to the core idea as well as the experiment design and results analysis. YL and RW has provided assistance in experiments and analysis, under XZ's supervision. Besides, XZ provided the research group with financial support and experimental equipments, as well as being a supportive corresponding author.

## Conflict of Interest

The authors declare that the research was conducted in the absence of any commercial or financial relationships that could be construed as a potential conflict of interest.
